# Association between prolonged Emergency Department stay and Intensive Care Unit mortality among patients at a tertiary hospital in Ethiopia

**DOI:** 10.1016/j.afjem.2026.100979

**Published:** 2026-05-14

**Authors:** Nardos B. Feleke, Chernet T. Mengistie, Biruk T. Mengistie, Mikiyas G. Teferi, Meron H. Biza, Hiwot Shewangizaw, Yonathan Asnake, Getaw W. Hassen, Lemlem Beza

**Affiliations:** a Emergency and Critical Care Medicine Department, College of Health Sciences, Addis Ababa University, Addis Ababa, Ethiopia; b School of Medicine, College of Health Sciences, Addis Ababa University, Addis Ababa, Ethiopia

**Keywords:** Emergency department boarding, ED length of stay, Intensive care unit mortality, Low- and middle-income countries, transfer

## Abstract

**Background:**

Prolonged boarding of critically ill patients in the Emergency Department (ED) is common in low-resource settings and may worsen outcomes. We evaluated whether extended ED length of stay (EDLOS) was associated with increased intensive care unit (ICU) and ED mortality at Tikur Anbessa Specialized Hospital, Addis Ababa, Ethiopia.

**Methods:**

We performed a retrospective cohort study of adults admitted from the ED to the ICU during 2023 (n = 110). EDLOS (triage to ICU admission) was categorized into four groups: <24 h, 48–72 h, and >72 h. ICU mortality by EDLOS group was calculated. Multivariable logistic regression was used to identify independent predictors of ICU death, adjusting for age, Charlson comorbidity index (CCI), mechanical ventilation, diagnosis category, coma, and systolic blood pressure <90 mmHg.

**Results:**

A total of 74 patients (67.3%) were admitted to the ICU within 24 h, and 36 (32.7%) were boarded >24 h. ICU mortality increased with longer EDLOS: 39.2% (<24 h), 58.8% (48–72 h), and 58.3% (>72 h). After adjustment, EDLOS of 48–72 h was associated with higher odds of ICU death compared with <24 h ((Adjusted Odds Ratio) AOR 3.34; 95% CI 1.22–9.16; p = 0.019), whereas EDLOS >72 h was not statistically significant. Other independent predictors of ICU mortality included ED systolic blood pressure <90 mmHg (AOR ≈ 7.0), respiratory admission diagnosis (AOR ≈ 3.9), and a one-point increase in Charlson comorbidity score (AOR ≈ 1.6).

**Conclusion:**

At this tertiary care center, prolonged ED boarding beyond 48 h was associated with substantially higher ICU mortality. Boarded patients had more comorbidities and were hemodynamically unstable. Our findings underscore the urgent need to enhance ICU access, prioritize timely transfers of the sickest patients, and strengthen ED critical care capacity in similar low-resource settings.

## African relevance

• This study quantifies the impact of prolonged ED boarding on ICU mortality in Ethiopia.

• Identification of hemodynamic instability and comorbidity are key as high-risk markers.

• Prioritising timely ICU transfer and ED critical care capacity is key in Africa.

## Introduction

Prolonged boarding of critically ill patients in the Emergency Department (ED) is recognized as a cause of ED crowding and poor outcomes. Prolonged Emergency Department Length of Stay (EDLOS) has multiple clinical implications [1,2]. Prolonged EDLOS is often defined by experts as an ED stay exceeding 4–6 h from ED arrival or triage, although definitions vary across studies [3,4]. Over the past two decades, ED crowding and prolonged EDLOS have become a challenge worldwide, straining resources and delaying definitive care [1,2]. Boarding could compromise timely interventions such as antibiotics or vasopressors in sepsis, which can worsen outcomes [5,6]. Overall, prolonged EDLOS is associated with increased Intensive Care Unit (ICU) mortality, longer hospital stays, higher complication rates, and patient dissatisfaction [2,5,7].

Definitions and thresholds for “prolonged EDLOS” vary by context [3]. International critical care groups (e.g., the Society of Critical Care Medicine) advocate that critically ill patients be admitted to the ICU within hours; the Surviving Sepsis Campaign now recommends ICU transfer within 6 h for septic shock [8]. Some Emergency Medicine organizations set a maximum boarding time of 4–6 h from ED arrival or triage, to ensure quality [3,4]. By contrast, Ethiopia and similar low-resource health systems commonly use 24 h as the threshold for prolonged ED stay [9,10]. We therefore define prolonged EDLOS as lasting ≥24 h in this setting, consistent with national norms.

Sub-Saharan Africa has very limited critical care capacity, so ED boarding is common [6,11]. Ethiopian guidelines stipulate that EDLOS should not exceed 24 h [9,10]. However, surveys found that the majority of critically ill patients in Ethiopia experience ED boarding beyond 24 h [6,9,12]. This underscores the magnitude of prolonged EDLOS in Ethiopia and similar low-and-middle-income countries (LMICs). By contrast, high-income settings report far lower rates [13].

Unlike the ICU, the ED is designed primarily for rapid assessment, short-term stabilization, and disposition, whereas the ICU provides continuous monitoring, closer nurse-to-patient ratios, and organ-supportive care for unstable patients. Prolonged EDLOS may therefore expose critically ill patients to worse outcomes [14,15]. Critically ill patients boarded in the ED routinely have higher mortality [16,17]. Delays in ICU transfer may contribute through delayed escalation of care, limited monitoring, and progression of organ dysfunction while awaiting definitive critical care [18]. Prolonged EDLOS also correlates with longer hospital stays, higher hospital charges, and increased risk of complications such as ICU-acquired infections [7,17,18]. In summary, boarding not only lengthens stay but also increases adverse events [2,7]. Families and patients also report lower satisfaction and perceived quality when critical care is delayed [19].

Multiple patient- and system-level factors may contribute to delayed ICU transfer and its associated adverse outcomes. Patients with greater illness severity, including comorbidities and hemodynamic instability, are at increased risk of mortality [16,18,20]. At the same time, system constraints such as limited ICU capacity and delays in transfer may prolong boarding and restrict access to continuous monitoring and definitive critical care [3,6,21]. Delays in escalation of treatment and ongoing resuscitation during prolonged ED stays may further contribute to clinical deterioration [5,14,16]. Together, these factors suggest that prolonged EDLOS may be associated with worse outcomes among critically ill patients [3,16,17].

Beyond illness severity and access constraints, outcomes may also be shaped by how ED care is organized. The ED is designed for rapid assessment and stabilization. By contrast, longitudinal critical care depends on ICU-level monitoring, staffing, and repeated reassessment. This mismatch can leave boarded patients exposed to delayed recognition of deterioration. Limited experience with ongoing critical-care management and imperfect handover/communication can further delay escalation and follow-up for unstable patients [3,14,22,23].

Despite international guidance to minimize ED boarding of critically ill patients, many tertiary hospitals in Ethiopia report prolonged ED stays and limited ICU capacity. Whether and how prolonged EDLOS affects ICU mortality in this context remains inadequately quantified. This study, therefore, aimed to determine the association between protracted ED length of stay and ICU mortality among critically ill adults at Tikur Anbessa Specialized Hospital, and to identify patient- and system-level predictors that could inform targeted triage and capacity-building interventions.

## Methods

This retrospective cohort study was conducted at Tikur Anbessa Specialized Hospital (TASH), a major tertiary referral and teaching hospital in Addis Ababa, Ethiopia. TASH is the main teaching hospital for Addis Ababa University College of Health Sciences and primarily serves the population of Addis Ababa while also receiving referrals nationwide. The hospital has approximately 700 beds, including a central intensive care unit (ICU) with six medical, four paediatric, and six surgical beds. The adult ED provides 24-hour services and manages an estimated 18,000–20,000 visits annually (50–55 per day). Data were collected from medical charts of patients admitted from the ED to the ICU in 2023. Data abstraction was performed between 15 May and 21 September 2024.

All adult patients (≥18 years) admitted from the ED to the adult ICU during the study period were eligible. Inclusion required complete data on ED triage time, ICU acceptance time, hospital outcome, and key clinical variables. We excluded patients who died in the ED or en route to the ICU, those transferred from other facilities after an ED stay, and those who left against medical advice. After identifying all eligible patients (sampling frame ≈137), we obtained 180 charts; 70 were excluded for missing or incomplete data, leaving a final sample of 110 patients.

The primary outcome was ICU mortality (death during the ICU stay). The secondary outcome was the total in-hospital length of stay (days from ED triage to hospital discharge or death). The main exposure was ED length of stay (EDLOS), defined as the time from ED triage to ICU admission. Prolonged EDLOS was defined as >24 h. For regression, EDLOS was categorised as <24 hours (reference), 48–72 hours, and >72 hours. No patients fell into a separate 24–48 hour category, so it was not analyzed independently. Independent variables included age, sex, primary diagnosis at ICU admission, Charlson comorbidity score, and need for mechanical ventilation (ED or ICU). Components of the Mortality Probability Model-0 (MPM₀) were also recorded.

Data were extracted using a structured form adapted from previous studies, with permission from the original source [24]. Variables included socio-demographics, clinical presentation, ED/ICU timestamps, interventions (mechanical ventilation, CPR), Charlson comorbidities, MPM₀ components, EDLOS, and outcomes. Four trained medical interns performed data abstraction following standardized training and supervised practice. The tool was pilot-tested on 10 charts and refined. Ongoing supervision, periodic spot-checks, and re-abstraction of 10% of charts were conducted to ensure data quality, with discrepancies resolved by consensus. Charts missing key variables were excluded, and the final dataset was checked for completeness and consistency.

Sample size was calculated using the single population proportion formula, n = Z²p(1 − p)/d², with a 95% confidence level, 5% margin of error, and an assumed ICU mortality of 35%, yielding n = 348. Applying finite population correction (N ≈ 137) resulted in an adjusted sample of 99.4. After adding 10% for incomplete data, the final sample size was 110.

Data were analysed using IBM SPSS Statistics version 26. Descriptive statistics were summarised by the EDLOS group (<24 hours vs. >24 hours). Continuous variables were reported as medians with interquartile ranges (IQRs) and compared using the Mann–Whitney U test. Categorical variables were reported as counts and percentages, and compared using chi-square tests. Spearman correlation assessed continuous associations. Variables with P < 0.25 in bivariate analysis were included in multivariable logistic regression. Adjusted odds ratios (AORs) with 95% confidence intervals (CIs) were reported, with statistical significance set at P < 0.05.

The study was approved by the Institutional Review Board of Addis Ababa University College of Health Sciences (IRB approval no AAU-CHS/PG/2024/041). All procedures followed the Declaration of Helsinki and relevant national research ethics guidelines.

## Results

### Participants

Of the 180 charts reviewed, 70 were excluded. As a result, a total of 110 patients were included in the analysis.

### Baseline characteristics

Patients in the delayed-admission group were generally older and had a higher comorbidity burden than those admitted within 24 h. Mechanical ventilation in the ED was common in both groups. Respiratory diagnoses were more frequent among delayed admissions, whereas cardiovascular diagnoses were more common in the early-admission group. Total hospital length of stay was similar between groups. Coma or stupor and pre-ICU CPR were uncommon, while ICU mortality was higher in the delayed group (Table 1).

**Table 1:** Sociodemographic patterns of patients admitted early and late.

Variables	Overall (N = 110)	Early EDLOS <24 hourrs (n = 74)	Late EDLOS >24 h (n = 36) [48–72 h (n = 24), >72 h (n = 12)]	P-value
Age, median (IQR), years	-	38(25–50)	52(37–71)	0.455
Sex – n (%)				0.165
Male	47(42.7)	35(47.3%)	12(33.3%)	
Female	63(57.3)	39(52.7%)	24(66.7%)	
Charlson score, median (IQR)	-	1(0–2)	2(1–4)	0.637
Mechanical ventilation – n (%)	51(46.4)	34(45.9%)	17(47.2%)	0.900
Main reason for admission – n (%)				0.060
Respiratory	39(35.4)	24(24%)	22(44.9%)	
Cardiovascular	32(29.2)	29(29%)	9(18.4%)	
Neurological	19(17.7)	13(17.6%)	3(8.3%)	
Airway protection	20(18.2)	8(10.8%)	4(11.1%)	
Total in-hospital length of stay	-	12(6–22)	11(7–18)	0.403
MPM score				
Coma	28(25.5)	19(25.7%)	9(25%)	0.939
SBP < 90mmHg	35(31.8)	22(29.7%)	13(36.1%)	0.500
AKI	37(33.6)	22(29.7%)	15(41.7%)	0.214
CPR before admission	6(5.5)	5(6.8%)	1(2.8%)	0.389
Outcome of patients				0.058
Alive	60(54.5)	45(60.85%)	15(41.7%)	
Dead	50(45.5)	29(39.2%)	21(58.3%)	

**Footnote:** Abbreviations: ED, emergency department; EDLOS, emergency department length of stay; ICU, intensive care unit; IQR, interquartile range; AKI, acute kidney injury; CPR, cardiopulmonary resuscitation; SBP, systolic blood pressure; MPM₀, Mortality Probability Model at admission.

### Primary outcome: ICU mortality

**Fig. 1:**
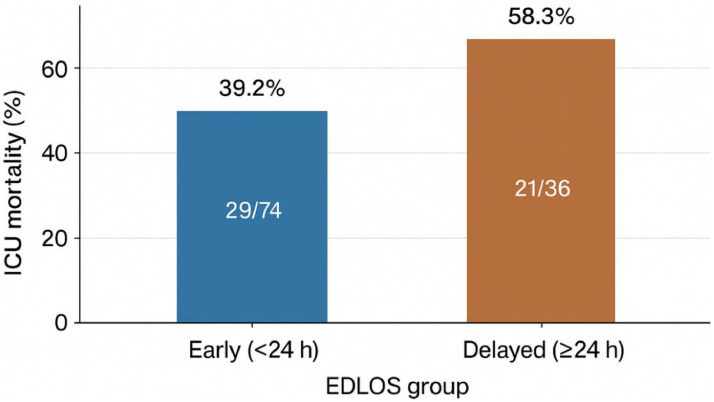
Intensive Care Unit (ICU) mortality by Emergency Department Length of Stay (EDLOS) category.

In unadjusted analysis, ICU mortality was higher among delayed admissions compared with early admissions (Fig. 1), with a crude odds ratio of 2.17 (95% CI 0.97–4.89). This difference was statistically significant (p < 0.05, χ² test).

Variables with p < 0.25 in bivariate analysis were included in the multivariable logistic regression model. After adjustment, EDLOS remained an independent predictor of ICU mortality. Specifically, EDLOS of 48–72 h was associated with higher odds of ICU death compared with <24 h, whereas EDLOS >72 h was not statistically significant. Other independent predictors of ICU mortality included hypotension on ED arrival, respiratory admission diagnosis, and a higher Charlson comorbidity score, while age and mechanical ventilation were not independently associated (Table 2).

**Table 2:** Association of EDLOS (using 3 categories) and ICU mortality (Bivariate and multivariate regression).

Variables	COR(95% CI)	P-value	AOR(95% CI)	P-value
Age	-	-	1.022(0.98–1.06)	0.283
EDLOS				
<24 h (reference)	1		1	0.019
48–72 h	4.050(1.030–15.924)	0.045	3.344(1.22–9.155)	
>72 h	1.211(0.473–3.098)	0.69		
Mechanical ventilator in the 1st 1 hr				
No(Reference)	-			
Yes			2.244(0.586–8.437)	0.240
AKI	-			
No (Reference)				
Yes			1.522(0.512–4.524)	0.449
SBP<90	-			
No (Reference)				
Yes			7(1.691–29.7)	0.007
Coma or stupor	-			
No (Reference)				
Yes			4.003(0.835–19.19)	0.083
The main reason for admission(respiratory)	-			
No (Reference)				
Yes			3.906(1.055–14.462)	0.043
The main reason for admission(cardiovascular)	-			
No (Reference)				
Yes			2.054(0.704–6.393)	0.410
Charlson score	-	-		
0(reference)				
>0			1.612(1.094–2.373)	0.016

*Abbreviations*: AOR, adjusted odds ratio; CI, confidence interval; COR, crude odds ratio; EDLOS, emergency department length of stay; ICU, intensive care unit; SBP, systolic blood pressure; AKI, acute kidney injury; CPR, cardiopulmonary resuscitation.

Variables, including age and need for mechanical ventilation, were not statistically significant in the adjusted model (Table 3).

**Table 3:** Association of other confounding factors and ICU mortality (Bivariate and multivariate regression).

Variables	COR(95% CI)	P-value	AOR(95% CI)	P-value
Age	1.014(0.993–1.035)	0.187	0.979(0.94–1.018)	0.283
EDLOS				
<24 h (reference)	1		1	0.019
48–72 h	4.050(1.030–15.924)	0.045	3.344(1.22–9.155)	
>72 h	1.211(0.473–3.098)	0.69		
Mechanical ventilator in the 1st 1 hr				
No(Reference)				
Yes	4.529(2.026–10.126)	<0.001	2.244(0.586–8.437)	0.240
AKI				
No (Reference)				
Yes	1.991(0.893–4.44)	0.092	1.522(0.512–4.524)	0.449
SBP<90				
No (Reference)				
Yes	2.379(1.047–5.405)	0.038	7(1.691–29.7)	0.007
Coma or stupor				
No (Reference)				
Yes	2.812(1.153–6.858)	0.023	4.003(0.835–19.19)	0.083
The main reason for admission(respiratory)				
No (Reference)				
Yes	4.895(2.162–11.081)	<0.001	3.906(1.055–14.462)	0.043
The main reason for admission(cardiovascular)				
No (Reference)				
Yes	0.345(0.149–0.799)	0.013	2.054(0.704–6.393)	0.410
Charlson score				
0(reference)				
>0	1.288(1.066–1.556)	0.009	1.612(1.094–2.373)	0.016

*Abbreviations*: AOR, adjusted odds ratio; CI, confidence interval; COR, crude odds ratio; EDLOS, emergency department length of stay; ICU, intensive care unit; SBP, systolic blood pressure; AKI, acute kidney injury; CPR, cardiopulmonary resuscitation.

### Secondary outcome: Total in-hospital length of stay

**Fig. 2:**
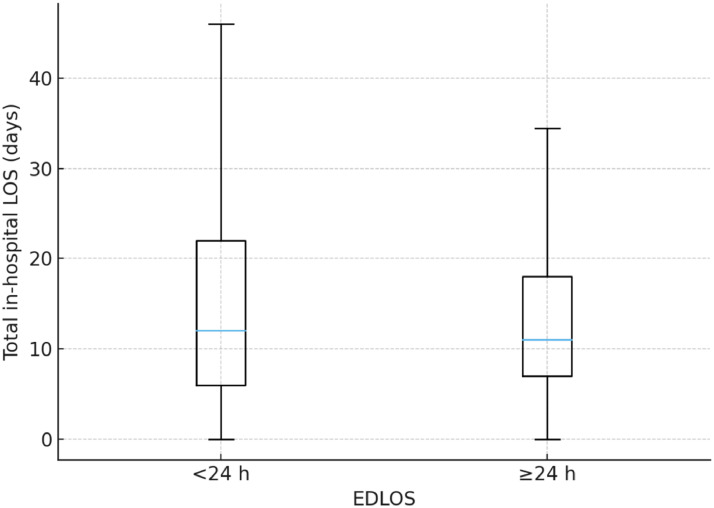
Distribution of total in-hospital length of stay by emergency department length of stay (EDLOS <24 h vs ≥24 h). *Boxes represent the median and interquartile range (IQR). For patients with EDLOS <24 h, the median was 12 days (IQR 6–22); for those with EDLOS ≥24 h, the median was 11 days (IQR 7–18). Mann–Whitney U = 1279.5, Z = −0.335, P = 0.738, R = 0.03 (negligible effect).*

The median total hospital length of stay did not differ significantly between groups. It was 12 days (IQR 6–22) for early admissions and 11 days (IQR 7–18) for delayed admissions (Fig. 2). The difference was not statistically significant. In line with this, none of the examined confounders (age, Charlson score, mechanical ventilation, diagnosis, coma, and SBP<90) showed a significant association with total hospital LOS.

### Additional analyses and missing data

All analyses included the whole cohort of 110 patients. We did not perform any subgroup or sensitivity analyses beyond those described. No participants had missing data for key variables after initial exclusion of incomplete charts.

## Discussion

Our main finding is that prolonged ED boarding was associated with higher ICU mortality, especially when EDLOS reached 48–72 h. The lack of a clear mortality signal in the >72-hour group is likely due to the very small number of patients in that category. Most unstable patients may have been transferred earlier or died before prolonged boarding could be observed. In this cohort, prolonged boarding was common. One third (32.7%) of patients waited >24 h for ICU admission, consistent with reports from other African settings [11,12,25]. These delays are far beyond the 4–6 h targets used in many high-resource systems [4,16], reflecting critical care scarcity. Our findings, therefore, suggest that delay itself, not only illness severity, contributes to worse outcomes.

Similar to our findings, prior studies have reported increased mortality with prolonged EDLOS. Evidence from Saudi Arabia and Taiwan supports this association, whereas one Canadian study found no independent effect after adjustment, suggesting that resource context may modify the impact of boarding [7,13,17]. These differences likely reflect resource context: high-income hospitals may mitigate boarding by providing ICU-level care in ED, whereas under-resourced EDs may lack staff/equipment [3,26].

Several mechanisms likely explain our finding of higher mortality with prolonged EDLOS. First, boarding delays definitive ICU interventions. Critically ill patients often require time-sensitive therapies (e.g., early fluids/antibiotics in sepsis or thrombolysis in stroke) [8,16,21]. Delays in these therapies allow disease progression [5]. Second, EDs often lack critical care nurse staffing and continuous monitoring, so boarded patients may experience unmonitored deterioration [3,27]. Boarding has also been linked to more medical errors and less frequent reassessment [3,27,28]. Third, EDLOS itself can be a marker of system stress: high ED occupancy is tied to worse outcomes [7,28]. In our study context, prolonged boarding mainly resulted from ICU bed shortages (as noted in prior Tikur Anbessa reports [6]); hence, boarded patients likely waited without advanced care, increasing mortality.

Among patient factors, those boarded longer were older and had more comorbidity, but the most important predictors of ICU mortality were comorbidity burden and hemodynamic instability. Indeed, older age is a well-established predictor of critical illness mortality, and was higher among longer-boarded patients here [29,30]. Although age was not significant in multivariate analysis, it correlated with frailty and comorbidities (higher Charlson score), which remained significant. By contrast, the Charlson comorbidity index remained an independent predictor, with each point increasing the odds of death by about 1.6 times. This is consistent with prior studies showing that chronic illnesses increase ICU mortality and worsen critical care outcomes [[31], [32], [33], [34]].

Hypotension on ED arrival was the single strongest predictor of ICU death in our study (AOR≈7). This is biologically plausible since it indicates shock and end-organ hypoperfusion [8,20]. Numerous sepsis and shock cohorts and guidelines emphasize that initial circulatory failure confers markedly worse outcomes and that delays in definitive resuscitation increase death risk [8,16]. The independent association of respiratory admission likely reflects the severity of acute respiratory failure in this cohort [3,29]. Mechanical ventilation itself was not an independent predictor after adjustment in our model. This may reflect collinearity: it commonly co-occurs with other markers of physiologic severity, so the variance it explains may be captured by those other variables in the model rather than appearing as an independent effect [17,35].

The MPM₀ findings should be interpreted cautiously. In our cohort, predicted mortality at ICU admission was high, and a small increase in MPM₀ was associated with higher mortality. This aligns with prior evaluations showing that MPM₀ and other ICU-based models, such as SAPS II and APACHE, demonstrate only fair discrimination when applied to emergency or mixed critical care cohorts**,** with reported AUCs generally in the 0.65–0.75 range [[36], [37], [38]]. Recent external validations confirm that MPM₀ variants often perform suboptimally outside ICU settings, reflecting limited calibration for emergency populations [[37], [38], [39]]. These models were designed for patients already in the ICU rather than for ED triage, and their accuracy decreases when data elements are missing or delayed, which is common in low-resource environments [37,39]. Therefore, MPM₀ should be viewed as supportive rather than definitive in this setting.

Clinically, our findings highlight the urgent need to improve ICU triage and boarding protocols in Ethiopia and similar LMICs. Hospitals should prioritize early identification of high-risk patients (e.g., hypotensive, multi-comorbid) and expedite their transfer or equip EDs to provide ICU-level care temporarily [16,27]. Staffing and training of ED personnel in critical care skills may mitigate some boarding risks. At the policy level, investment in ICU capacity is essential [40]. In the meantime, creative solutions, such as a “critical emergency unit” (as trialed in Italy) or an ED-based ICU model, may be helpful [1]. Finally, during surge events (e.g., epidemics), flexible ICU triage plans and intermediate care units should be emphasized to reduce ED boarding [41,42]. Efforts to reduce preventable deaths should focus on timely ICU admission, earlier identification of high-risk patients, and better critical care support for boarded patients in the ED [3,17,28,43].

### Limitations

This study has several significant limitations. Its retrospective, single-center chart-review design risks missing data and misclassification, and the relatively small sample size limits statistical power and generalisability. A relatively large proportion of reviewed charts were excluded because of missing or incomplete key variables, which may have introduced selection bias and reduced the generalisability of the findings. Unmeasured clinical or system factors may confound the observed association between EDLOS and ICU mortality. Our use of a 24-hour EDLOS cutoff reflects local practice but differs from many international thresholds, limiting comparability. The MPM₀ was applied for risk adjustment; however, its performance in this setting is uncertain and may be affected by incomplete data. In addition, the relatively small sample size, particularly in the >72-hour EDLOS subgroup, may have limited statistical power for subgroup comparisons and contributed to imprecision in the estimated effects. Finally, EDLOS was derived from chart timestamps, which may introduce measurement error, and post-discharge outcomes were not captured.

## Conclusion

In this single-center Ethiopian cohort, prolonged ED stays were common and associated with excess ICU mortality: patients boarded beyond 48 h experienced markedly higher death rates than those admitted within 24 h. The association appears to be driven in part by a greater comorbidity burden and initial hemodynamic instability among delayed patients. These results support targeted interventions in Ethiopia and comparable LMIC contexts, increasing ICU capacity where feasible, implementing triage protocols that prioritize hypotensive and multi-morbid patients for expedited ICU admission, and equipping EDs to deliver short-term ICU-level care (through training, staffing, or intermediate/ED-based critical care units) while definitive ICU beds are unavailable. Monitoring EDLOS and outcome metrics should be part of quality improvement efforts aimed at reducing preventable ICU deaths.

## Dissemination of results

The findings of this study were shared with the clinical staff and administration of Tikur Anbessa Specialized Hospital Emergency Department. Results will also be presented at national and regional emergency medicine meetings to facilitate knowledge sharing and support evidence-based emergency care in similar resource-limited settings.

## Data availability

The datasets generated and/or analyzed during the current study are available from the corresponding author upon reasonable request.

## Funding

This research did not receive any specific grant from funding agencies in the public, commercial, or not-for-profit sectors.

## CRediT authorship contribution statement

**Nardos B. Feleke:** Conceptualization, Methodology, Formal analysis, Investigation, Writing – original draft. **Chernet T. Mengistie:** Conceptualization, Methodology, Writing – original draft, Writing – review & editing, Formal analysis. **Biruk T. Mengistie:** Methodology, Writing – original draft, Formal analysis. **Mikiyas G. Teferi:** Methodology, Writing – review & editing, Visualization. **Meron H. Biza:** Investigation, Writing – original draft. **Hiwot Shewangizaw:** Data curation, Writing – original draft. **Yonathan Asnake:** Writing – review & editing, Validation, Data curation. **Getaw W. Hassen:** Writing – review & editing, Methodology, Supervision, Project administration. **Lemlem Beza:** Writing – review & editing, Investigation, Resources, Supervision.

## Declaration of competing interest

The authors declared no conflicts of interest.
